# Buried penis repair: tips and tricks

**DOI:** 10.1590/S1677-5538.IBJU.2020.99.06

**Published:** 2020-01-20

**Authors:** Jacob Robert Stephen, Frank N. Burks

**Affiliations:** 1 Department of Urology Oakland University William Beaumont School of Medicine Royal OakMichigan USA Department of Urology, Oakland University William Beaumont School of Medicine, Royal Oak, Michigan, USA

**Keywords:** Penis, Obesity, Urethral Stricture

## Abstract

Obesity is increasing in prevalence worldwide and an increasingly commonly encountered condition is adult acquired buried penis (AABP). We review the current management of AABP and relevant literature. Management of AABP requires a combination of genitourinary reconstructive techniques and plastic surgery techniques that are unique to this condition. We offer our experience and tips and tricks for the treatment of AABP.

## INTRODUCTION

Obesity is increasing in prevalence worldwide, with rates nearly tripling from 1975-2016 ( 1 ). This is associated with numerous comorbidities, including type 2 diabetes mellitus, obstructive sleep apnea, coronary artery disease, stroke, and various cancers, to name a few ( 2 ). Unfortunately, the genitourinary system is not spared, as obesity is also associated with adult acquired buried penis ( 3 ). Adult acquired buried penis is not a benign condition; it causes significant psychosocial distress. In addition, a recent study suggested that it was associated with a higher incidence of penile carcinoma ( 4 ). Adult acquired buried penis is also associated with concomitant urethral strictures, with rates as high as 31-47% ( 5 , 6 ), likely due to associated lichen sclerosis and chronic inflammation. These can complicate repair and often demand their own attention either prior to or coincident with buried penis repair.

While obesity is not the sole etiology of adult acquired buried penis, as it can also result from cicatrix formation due to overzealous circumcision, penile skin loss resulting from lichen sclerosis, or, rarely, pelvic lymphedema, it remains one of the most significant risk factors for the development of this condition. Unfortunately, weight loss alone is often ineffective and definitive treatment requires surgical repair.

Operative techniques have been previously documented in the literature ( 7 - 10 ), but typically include the following general steps: dorsal slit to expose the glans followed by degloving of the diseased penile skin, escutcheonectomy with or without panniculectomy, and harvest and application of split thickness skin graft Jun et al. ( 9 ) described their operative technique in a previously published article. Some notable items from their technique include using the anterior thigh for their split thickness skin graft (as opposed to using the resected skin from the panniculectomy/escutcheonectomy (8, 10) or other harvest sites) and securing their grafts with fibrin sealant after suturing proximally and distally Pariser at al. ( 5 ) recently proposed a classification system to stratify buried penis repair by complexity of repair. This includes the following categories: Category I – penile unburying with local skin flap; Category II – use of skin graft; Category III – scrotal surgery; Category IV – escutcheonectomy; and Category V – abdominal panniculectomy. When reviewing outcomes based on classification, more complex repairs (i.e. Category III-V) were associated with higher incidence of high-grade complications including wound dehiscence, abscess requiring operative intervention, and scrotal hematoma, among other complications.

In this article, we review the current literature on adult acquired buried penis repair as well as offer several tips and tricks we use in our practice.

## DISCUSSION

### Pre-operative Evaluation

Our pre-operative evaluation generally consists of a comprehensive medical and surgical history as well as a physical examination. Given the aforementioned co-morbidities often associated with adult acquired buried penis, it is imperative that patients’ other medical issues are optimized prior to any surgical intervention. Additional evaluations include assessing baseline erectile function and voiding symptoms. In our practice, all patients are administered the American Urologic Association Symptom Index. Because of the high rates of comorbid urethral stricture disease ( 5 , 6 , 11 ), if patients are found to have symptoms suggestive of a stricture, further evaluation is performed. This can include either a pre-operative retrograde urethrogram or intra-operative urethroscopy. Often a perineal approach such as a Kulkarni urethroplasty is required for longer segment or proximal strictures, and these are usually performed prior to a buried penis repair with at least six months between the two procedures to allow appropriate healing. More distal strictures can be managed at the time of buried penis repair.

**Tip #1:** We recommend screening all AABP patients for potential urethral stricture using validated voiding symptom questionnaires such as the AUA-SI and performing either urethroscopy or retrograde urethrogram as indicated;

**Tip #2:** Should a patient require a urethroplasty prior to buried penis repair, we recommend allowing at least 6 months between surgeries.

### Management of Cicatrix, Escutcheonectomy and/or Panniculectomy

Although most commonly the penile skin is diseased or obliterated due to lichen sclerosis or chronic inflammation, there are rare instances in which the penile skin is salvageable. These typically result from overaggressive circumcision. In these cases, one described technique for unburying is a ventral slit with scrotal flap ( 12 ). In this technique, a ventral slit is made to expose the glans, the incision is carried down the median scrotum from midshaft to the mid scrotum, and a relaxing incision is carried from the mid scrotum horizontally to create a rotational flap. The ventral penile skin defect is then covered using the scrotal skin before closing the scrotum. With this technique, the dorsal penile skin is viable and maintained.

However, the majority of cases of adult acquired buried penis are the result of morbid obesity and chronic inflammation, and in many of these cases the penile skin is nonviable. In these cases, the penile skin is completely degloved, taking extreme care to avoid leaving a remnant of skin near the corona, as any remnant can become an edematous ring of tissue. The escutcheon is resected to the level of the abdominal wall fascia. If there is any lymphedematous tissue, the entirety of the diseased skin, underlying dermis, and Dartos tissue should be resected to ensure removal of the lymphatics and prevent reburying. One additional important step is to secure the remaining suprapubic flap to the pubic bone or Buck’s fascia in order to prevent disease recurrence. We often place drains to promote healing, although not all surgeons do the same ( 13 ).

**Tip #3:** Ensure that the entirety of the penile skin is removed, particularly near the corona to avoid leaving an edematous ring of diseased tissue;

**Tip #4:** To avoid recurrence of adult acquired buried penis due to lymphedema, it is imperative that the entirety of the diseased skin, dermis, and dartos tissue is resected to remove the lymphatics;

**Tip #5:** When performing escutcheonectomy, ensure that the skin is secured to Buck’s fascia or the pubis to prevent re-burying.

### Harvest and application of split thickness skin graft

Typically, we prefer to harvest our skin graft from the lateral thigh. However, others have reported successful outcomes when using sections of the resected escutecheon or pannus ( 8 , 10 ), though care should be taken to ensure that the used segments are free of any lymphedema or signs of chronic inflammation.

When applying the graft, the penis is held on stretch, usually with the assistance of retention sutures placed through the glans at the start of the procedure. To prevent a cleft from forming at the base of the penis, it is helpful to advance a collar of scrotal skin and escutcheon skin around the base of the penis and secure the proximal end of the graft to this collar. Holding the penis on stretch while securing the graft proximally to Buck’s fascia and distally to the corona ensures that the graft will not fold on itself and will be appropriately apposed to the underlying tissue Iblher et al. ( 14 ) reported success with adjuncts such as intracavernosal prostaglandin injections or daily tadalafil post-operatively to promote penile engorgement and prevent graft contracture.

**Tip #6:** Apply the split thickness skin graft to the penis while on full stretch;

**Tip #7:** To prevent a cleft from forming at the base of the penis, advance a collar of scrotal and/or escutcheon skin around the penile shaft at the base and secure the split thickness skin graft to this collar.

While not typically used in our repairs, wound vacuums offer several benefits for healing and graft take. These include promoting microcirculatory flow and stimulation of angiogenesis, preventing graft lift by constant evacuation of fluid, exudate, and blood, and preventing graft shear ( 15 ).

### Post-operative care

At our institution, patients are typically admitted for 48 hours with foley catheter removal on post-operative day 2. Dressings are typically taken down on post-operative day 2 and drains are removed within the first 5-7 days. However, a recent case series published by Erpelding et al. ( 13 ) demonstrated the feasibility of same day discharge for patients undergoing buried penis repair. In their series, 10/16 patients were discharged same day with no differences in complication rate compared with those patients kept overnight. These patients were typically discharged with a foley catheter and a penile bolster dressing, both of which were then removed at a post-operative visit one week later. Interestingly, complexity of repair did not influence whether patients were kept overnight.

Despite the invasiveness of a buried penis repair, most patients, when asked, would have the surgery performed again due to their significant improvements in sexual function, voiding function, and overall quality of life ( 8 , 16 , 17 ). Theisen et al. ( 16 ) reported significant patient-reported in sexual domain and urinary domain, with 87.5% reporting improvement in overall urinary bother and 94% reporting improvement in their overall ability to function sexually. Similarly, a retrospective review by Hampson et al. ( 8 ) showed improvements in erectile function, sexual activity, genital hygiene, and ability to stand while urinating, among other improved functional outcomes. Importantly, these improvements were sustained at a mean of 39.4 months of follow-up. In addition, 85% of patients in their series stated that they would undergo the procedure again. Rybak et al. ( 17 ) quoted a 91% improvement in voiding erectile dysfunction, and quality of life, respectively, in patients undergoing buried penis repair. Thus, a properly performed buried penis repair achieves sustainable, satisfactory results and can have a significant impact on a patient’s overall well-being.

## CONCLUSIONS

As the incidence of obesity increases, so to will our encounters with patients who have developed adult acquired buried penis. Definitive treatment typically requires surgical repair to unbury the phallus and can lead to significant improvement in a patient’s quality of life. It is imperative that the reconstructive urologist be comfortable with surgical techniques involved in buried penis repair. We offer several of our own tips and tricks to assist in achieving successful patient outcomes
